# Improved Kerogen Models for Determining Thermal Maturity and Hydrocarbon Potential of Shale

**DOI:** 10.1038/s41598-018-35560-8

**Published:** 2018-11-30

**Authors:** Vikas Agrawal, Shikha Sharma

**Affiliations:** Department of Geology and Geography, West Virginia University, Morgantown, WV United States

## Abstract

Kerogen is the insoluble component of organic-rich shales that controls the type and amount of hydrocarbons generated in conventional and unconventional reservoirs. Significant progress has recently been made in developing structural models of kerogen. However, there is still a large gap in understanding the evolution of the molecular components of kerogen with thermal maturation and their hydrocarbon (HC) generative potential. Here, we determine the variations in different molecular fragments of kerogen from a Marcellus Shale maturity series (with VRo ranging from 0.8 to 3) using quantitative ^13^C MultiCP/MAS NMR and MultiCP NMR/DD (dipolar dephasing). These molecular variations provide insight into the (1) evolution of the molecular structure of kerogen with increasing thermal maturity and, (2) the primary molecular contributors to HC generation. Our results also indicate that old model equations based on structural parameters of kerogen underestimate the thermal maturity and overestimate the HC generation potential of Marcellus Shale samples. This could primarily be due to the fact that the kerogen samples used to reconstruct old models were mostly derived from immature shales (VRo <1) acquired from different basins with varying depositional environments. We utilized the kerogen molecular parameters determined from the Marcellus maturity series samples to develop improved models for determining thermal maturity and HC potential of Marcellus Shale. The models generated in this study could also potentially be applied to other shales of similar maturity range and paleo-depositional environments.

## Introduction

The advent of unconventional shale gas drilling has necessitated the need to develop a better understanding of the spatiotemporal variations in type and quality of organic matter in shale source rocks. Kerogen is a high molecular weight organic matter (OM) that serves as source and reservoir of all the hydrocarbons in these shales. Kerogen is formed by the degradation, condensation, and polymerization of biomolecules contributed by different sources of OM^1–3^. Kerogen formed in the diagenetic stage of burial later cracks to form oil and gas in catagenetic and metagenetic stages of burial. The type and amount of HC generated, its sorption/retention and release on hydraulic fracturing operations is controlled by the molecular structure of kerogen^3–6^. Therefore, it is critical to understand the chemical structure of kerogen at the molecular level. In addition, molecular parameters of kerogen can serve as a more robust proxy for determining thermal maturity and hydrocarbon potential in mature shales (with VRo >1) where traditional techniques such as vitrinite reflectance, SRA, and biomarker analysis fails.

Significant progress has been made in understanding the molecular structure of kerogen using destructive (pyrolytic) and non-destructive (spectroscopic) methods^7–10^. However, the results generated using pyrolytic experiments can be biased due to the interaction of products generated from the labile fraction of kerogen^10^ or because reactions taking place in laboratory conditions might not be representative of sedimentary basin conditions^11–14^. Due to these limitations, non-destructive methods such as Fourier transform infrared (FT-IR), Raman spectroscopy (RS), X-ray photoelectron spectroscopy (XPS), X-ray absorption near edge structure (XANES), and ^13^C solid-state nuclear magnetic resonance (^13^C NMR) have been employed for the qualitative, semi-quantitative, and quantitative measurements of kerogen^7,15–21^.

The most reliable and robust tool for determination of fractions of molecular components of kerogen is ^13^C solid state NMR^22^. Numerous studies have been conducted using ^13^C solid-state NMR for kerogen characterization^15–17,19,23–29^. A few recent attempts have been made to develop realistic structural models of kerogen^30,31^. However, the evolution of different molecular components of kerogen on thermal maturation and the primary contributors to HC generation are still not well understood, especially for mature shales. A recent study by Agrawal and Sharma, 2018^19^ indicated that kerogen structural parameters used in previous models for determining HC generative potential^28^ and thermal maturity^26,32^ could over or under estimate these values.

In this study, we determine the stability/reactivity of different molecular fragments of kerogen with thermal maturation and predict the primary contributors of hydrocarbons in a Marcellus shale maturity series (VRo ranging from 0.8 to 3). The variations in molecular parameters of kerogen were determined using quantitative MultiCP/MAS NMR and MultiCP NMR/DD (dipolar dephasing). Most of the previous investigations used CP/MAS NMR technique, a semi-quantitative technique that could not differentiate between non-protonated carbons (from protonated carbons) and mobile groups (from immobile groups). However, in this study, by using MultiCP/MAS NMR with and without dipolar dephasing, it was possible to determine the fraction of non-protonated carbons, protonated carbons, mobile groups and immobile groups (Supplementary Table 1). The correlation of structural components of kerogen with maturation and HC potential parameter (S2), was used to develop regression models (linear and multiple) for accurate estimation of thermal maturity and hydrocarbon generated. The structural parameters of 15 kerogen samples were determined using ^13^C multiple CP/MAS and multiple CP/MAS plus dipolar dephasing technique detailed in Agrawal and Sharma, 2018^19^. A total of 15 samples were obtained from six Marcellus shale wells (BG-1, WV-7, WV-6, MIP-3H, MW-1 and BL-3H) across a thermal maturity gradient in Appalachian basin (Table 1). TOC, S2 and VRo values of six samples were obtained from Agrawal and Sharma^19,33^ as indicated in Table 1.

**Table 1:** The TOC, S2 and VRo values of samples selected for from 6 different Marcellus Shale wells in the Appalachian basin.

Sample ID	TOC	S2	VRo	Sample ID	TOC	S2	VRo
BG-1 UM^i^	4.68	5.11	1	WV-6 LM^ii^	9.10	0.47	2.5
BG-1 LM^i^	15.40	15.84	0.81	BL-3H UM	5.31	0.06	2.93
WV-7 UM^ii^	3.13	1.41	1.4	BL-3H LM	9.24	0.15	2.96
WV-7 LM^ii^	12.91	10.67	1.4	MIP-3H UM	4.14	0.12	2.94
MW-1 UM	8.63	1.84	1.49	MIP-3H LM	8.86	0.42	2.98
MW-1 LM	7.45	1.38	1.61	MIP 3H MT	3.14	0.08	2.92
WV-6 UM^ii^	3.52	0.02	2.5	MIP 3H MM	6.64	0.30	2.96
				MIP 3H MO	5.35	0.26	2.97

^i^Values from *Agrawal and Sharma, 2018*^19^.

^ii^Values from *Agrawal and Sharma, 2018*^33^.

## Results and Discussion

**Figure 1:**
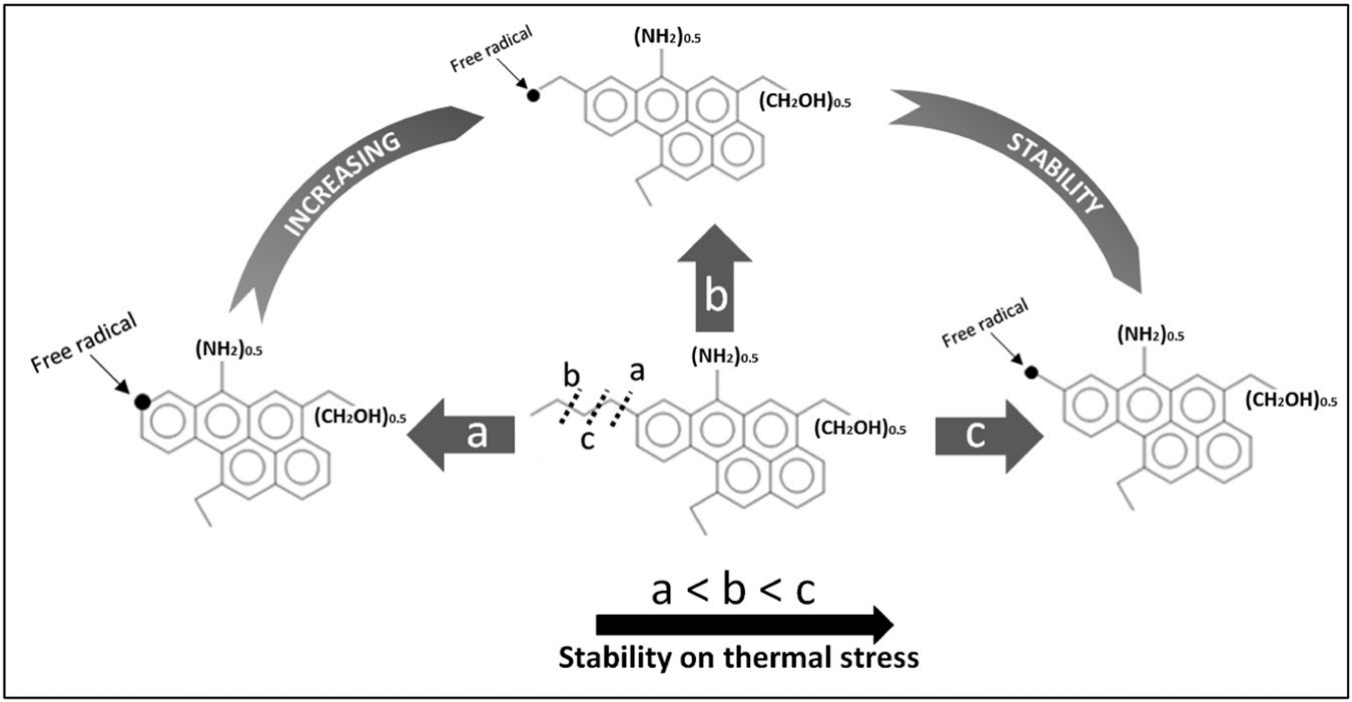
Stability of free radicals formed on thermal degradation. Structure of unit kerogen taken from Agrawal and Sharma, 2018^19^.

Different aliphatic and aromatic carbon chains have characteristic chemical shifts in an NMR spectra^34–37^ as shown in Table 1 of Supplementary Information. Dipolar dephasing method with multiple CP method allows the quantification of mobile aliphatic and protonated (and non-protonated) aromatic carbon chains along with other aliphatic and aromatic chains^8,22,38–40^ as shown in Table 1 of Supplementary Information. The aliphatic and the aromatic fraction in the NMR spectra lies in 0–90 ppm and 90–165 ppm chemical shift range respectively. The fractions of different aliphatic and aromatic chains of kerogen were calculated using the peak area of the respective chemical shifts in an NMR spectra. The fractions of aliphatic carbon, alkyl (without heteroatoms), methoxy and amine, O and O_2_ substituted alkyl carbons (ether and dioxy alkyl), total aromatic carbon, alkyl substituted aromatic, O-substituted aromatic (phenol), carboxyl and amide, aldehyde and ketone were determined using the multiple CP method (without dipolar dephasing). However, the fractions of mobile (freely rotating) and immobile (restricted rotation) methyl, mobile and quaternary alkyl (without heteroatoms), methoxy, protonated aromatic, non-protonated aromatic bridgehead carbon (fa^B^) were determined using dipolar dephasing method with multiple CP (Table 1 in the Supplementary Information). Using these aliphatic and aromatic structural parameters, several lattice structural parameters such as average aliphatic carbon chain length (Cn’), mole fraction of bridgehead aromatic carbon(Xb), and SP^2^/SP^3^ hybridized carbon ratio were determined^17,34,41^. The fractions of different structural parameters of 15 kerogen sample used in this study are shown in Table 1 of the Supplementary Information. Data of structural parameters of six samples are taken from Agrawal and Sharma^19^ (as indicated in Table 1 of the Supplementary Information). Multiple linear regression plots were made to determine the correlation of different structural parameters with VRo and S2. (refer to Figs 1–4 in Supplementary Information).

### Kerogen models for thermal maturity

Thermal maturity is one of the most important parameter required for accurate determination of the hydrocarbon generated by source rocks. The traditional methods used for determining thermal maturity are the Tmax measurement using Source Rock Analyzer (SRA) and vitrinite reflectance measurement. However, Tmax may not be reliable in mature and over-mature samples^42^, and vitrinite is present as a major maceral only in Type III kerogen. Errors are involved in measuring reflectance on macerals other than vitrinite^43^ or measuring reflectance of vitrinite whose thermal maturation is different from that of the bulk OM^3^. Biomarker ratios have also been used to determine the thermal maturity of source rocks^42^. However, due to thermal degradation and alteration of biomarkers on maturation^33,44^ and low extraction efficiency in high maturity samples, the results can be biased. Recent advancement in ^13^C solid-state NMR spectroscopic analysis, has led to an accurate quantification of different aliphatic, aromatic and lattice parameters of kerogen, even in over-mature shales^19^. Understanding the changes in these parameters on maturation can provide an important tool for determining thermal maturity in a broader maturity range.

In the Marcellus Shale the evolution of most of the structural parameters of kerogen such as immobile alkyl without heteroatoms (with restricted rotation), mobile (freely rotating), and alkyl-substituted aromatic carbons^19^ are primarily controlled by thermal maturity. Similar observations have also been made for shales from other basins basin^17,23,28^. However, the cracking mechanism of different structural parameters of kerogen can vary because of the difference in their thermal stability. To determine the sensitivity of different kerogen structural parameters with thermal maturation, linear correlation plots were made using the structural parameters of kerogen and calculated vitrinite reflectance (Figs 1 and 2 of Supplementary Information). It has been previously shown that maturation leads to a decrease in aliphatic and an increase in aromatic carbon chains^17,23,26,28^. However, the sensitivity and reactivity of carbon chains within the different aliphatic and aromatic carbon fractions are still not understood. Our results show that among different aliphatic chains, immobile alkyl chains are most prone to thermal degradation (R^2^ = 0.94), followed by mobile and quaternary alkyl carbon chains (R^2^ = 0.61) and mobile methyl carbon chains (R^2^ = 0.54, Fig. 1 of Supplementary Information). In the alkyl carbon chains (without heteroatoms) the immobile methyl group is most resistant to breakdown during maturation (R^2^ = 0.20). Immobile alkyl chains are mostly attached to the aromatic rings, restricting their rotation. The resistant nature of the immobile methyl group is possibly due to the instability of free radicals formed on thermal degradation (breaking of bond “a” in Fig. 1). The instability of the free radical is due to the disruption of resonance present the aromatic chains. However, for immobile alkyl groups, free radicals formed by thermal degradation (breaking of bond “c” in Fig. 1) is stabilized by the resonance of conjugated double bonds of the aromatic ring, making it the most stable amongst all the other aliphatic carbon chains.

In contrast, to the aliphatic structural parameters, the aromatic structural parameters showed relatively poor correlation to thermal maturation. Amongst the aromatic structural parameters, the highest correlation is observed between bridgehead aromatic carbons (R^2^ = 0.48) followed by protonated aromatic carbon (R^2^ = 0.06), O- substituted aromatic carbon (R^2^ = 0.06), and alkyl substituted aromatic carbon chains (R^2^ = 0.02). However, it is important to note that although individual aromatic parameters showed poor correlation with the vitrinite reflectance (Fig. 2 of Supplementary Information) the correlation of total aromatic with VRo is still significant (R^2^ = 0.90). This indicates that good correlation of total aromatic carbon with increasing maturity as determined by several previous studies is actually due to increase in the relative percentage of aromatic carbon due to the higher breakdown of aliphatic carbon chains rather than formation of new aromatic carbons. The similar mole fraction of bridgehead aromatic carbon (Xb) in all mature kerogen samples (VRo >1, Fig. 2 in Supplementary Information) also supports that the total amount aromatic clusters do not increase significantly with maturity. This observation is contrary to the previous studies done on lower maturity samples^26,32^.

**Figure 2:**
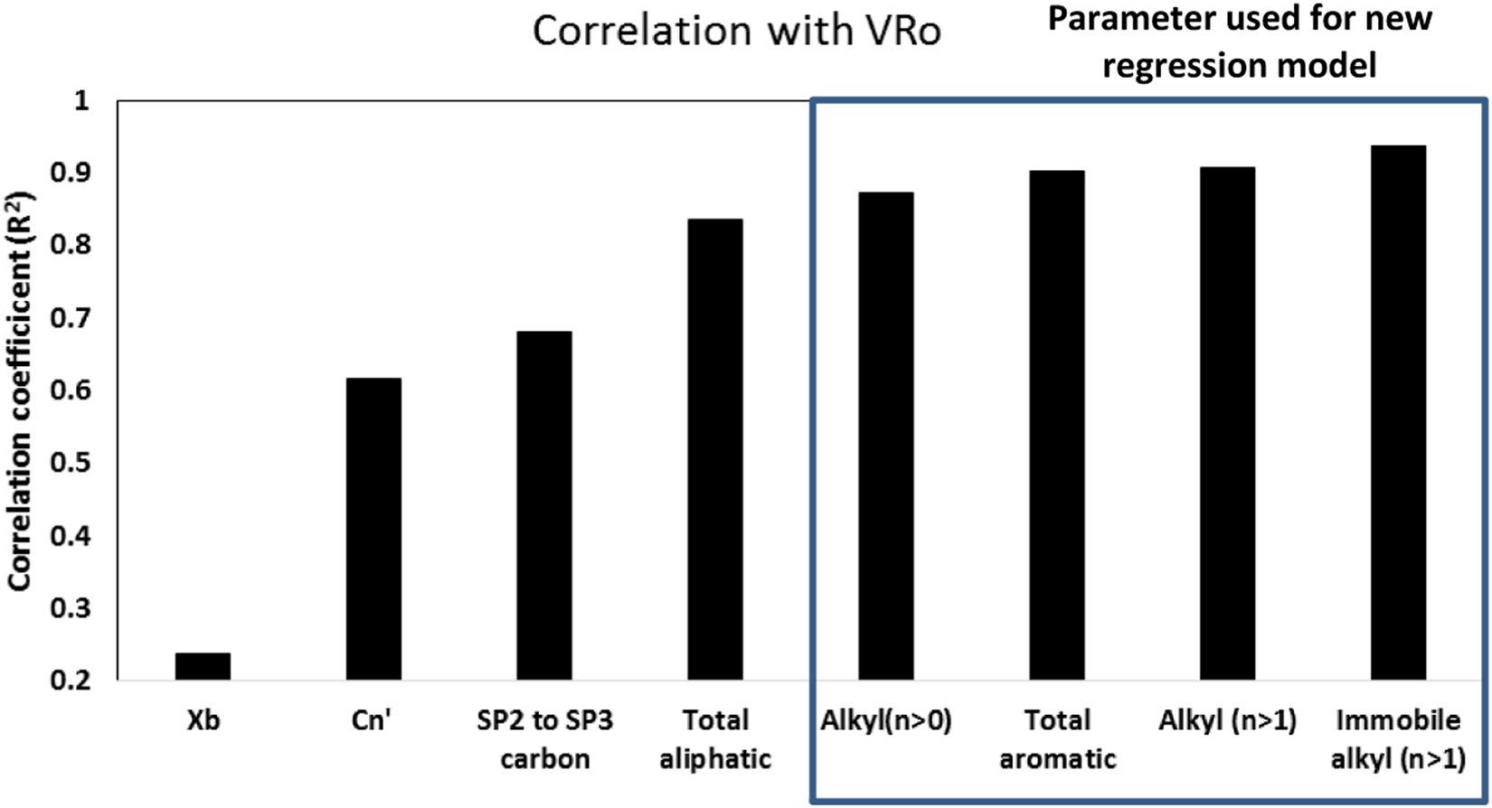
Correlation of different kerogen structural parameters with calculated vitrinite reflectance.

Regression models have been developed to determine thermal maturity using aromatic carbon percentage, the mole fraction of bridgehead carbon (Xb), SP^2^ to SP^3^ carbon ratio^26,32,45^. In this study, we observed that the correlation coefficient of immobile alkyl chains (CnH_2_n groups, with n > 1) was highest with R^2^ = 0.94 (95% confidence interval or CI of slope = ±2.99) followed by alkyl chains (immobile and mobile CnH_2_n groups, with n >1) with R^2^ = 0.91 (95% CI of slope = ± 2.82), then by total aromatic carbon (R^2^ = 0.90, 95% CI of slope = ±1.89) and by total alkyl (without heteroatoms) with R^2^ = 0.87 (95% CI of slope = ±2.54) as shown in Fig. 2. Based on these observations, we propose four new regression models for estimating thermal maturity in shales with VRo between 0.8 to 3.0 (all equations have R^2^ >0.85). The correlation coefficents and 95% confidence interval (CI) of the slope of the equations proposed for determining VRo were determined using software XLSTAT. The equations are as follows:

1 $${\rm{V}}{\rm{R}}{\rm{o}}=3.39-19.32\ast {\rm{I}}{\rm{A}}$$

where IA is fraction of immobile alkyl chains (CnH_2_n groups) with n >1.

2 $${\rm{VRo}}={\rm{3.41}}-{\rm{14.74}}\ast {{\rm{TA}}}_{{\rm{2}}}$$

where TA_2_ is fraction of total alkyl chains (CnH_2_n groups) with n >1.

3 $${\rm{VRo}}=-\,{\rm{5.84}}+{\rm{9.69}}\ast {{\rm{F}}}_{{\rm{ar}}}$$

where F_ar_ is fraction of total aromatic chains.

4 $${\rm{V}}{\rm{R}}{\rm{o}}=3.55\,-\,11.05\ast {\rm{T}}{\rm{A}}$$

where TA are total alkyl chains (CnH_2_n groups) with n >0.

### Kerogen models for hydrocarbon potential

**Figure 3:**
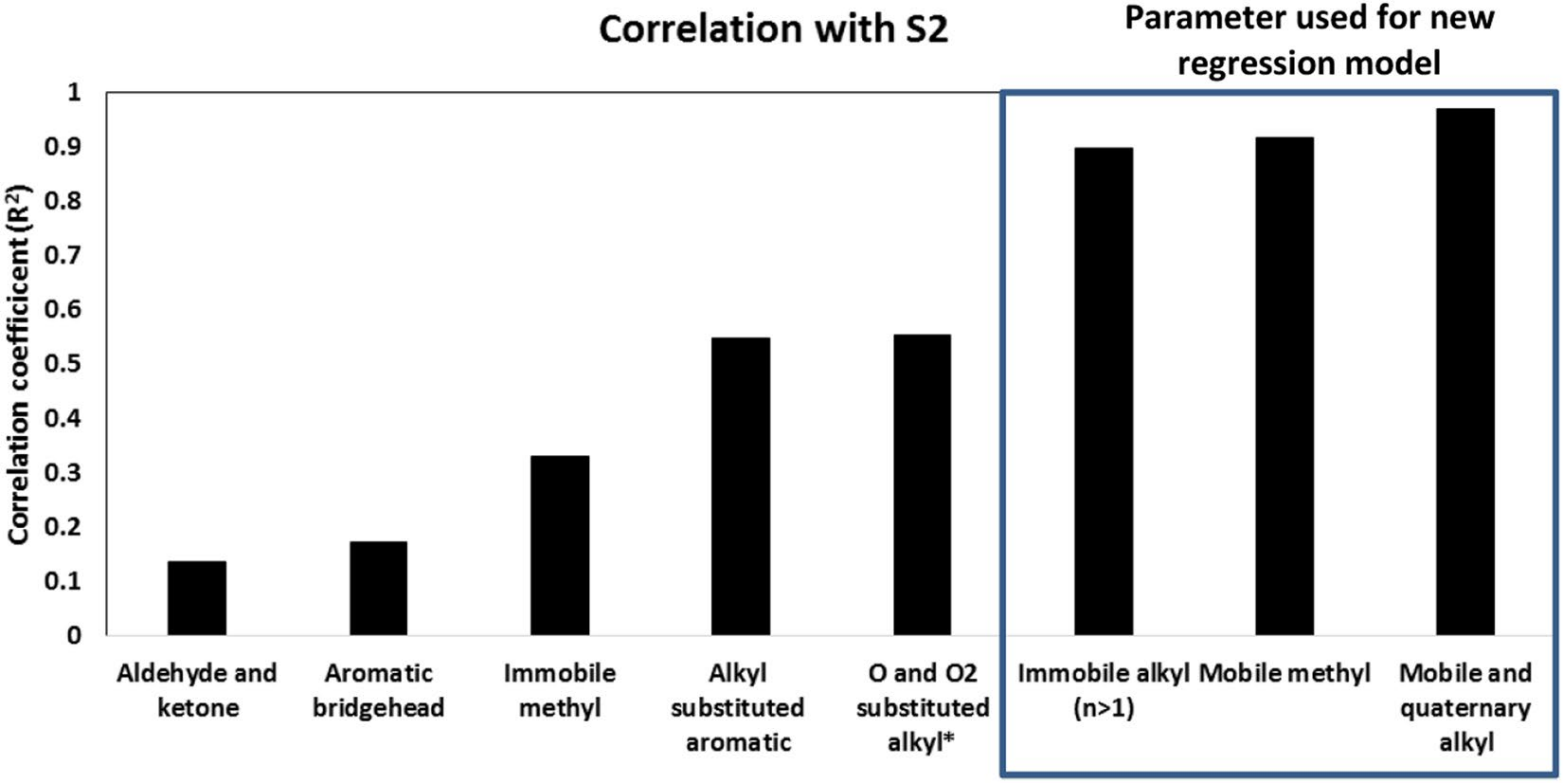
Correlation of different structural parameters of kerogen with true HC potential (S2).

A recent study done on kerogen from Marcellus Shale by Agrawal and Sharma, 2018^19^ indicates that previously build kerogen models used for determining HC generative potential overestimate the S2 values at least by a factor of two. This can lead to underestimation of the total amount of HC generated in the reservoir. We determined primary contributors to HC generation by evaluating the correlation of different structural parameters of kerogen with S2. We observed that the carbon chains that had highest correlation coefficient with S2 were mobile and quaternary alkyl group (R^2^ = 0.97, 95% CI = ±0.32), mobile methyl group (R^2^ = 0.92, 95% CI = ±0.55) and immobile alkyl chains (R^2^ = 0.90, 95% CI = ±0.17) (Fig. 3 in the Supplementary Information). Additionally, the relatively smaller correlation coefficient of immobile methyl, aromatic bridgehead carbon, aldehyde and ketone groups, and O and O_2_ substituted alkyl chains with S2 (Fig. 3), indicates there was little or no contribution from these structural components to the total hydrocarbons generation potential, contradicting the observations of Longbottom *et al*.^28^. Therefore, we propose a new equation for determining the hydrocarbon potential that is based on a multiple regression model using the fractions of structural parameters: mobile and quaternary alkyl group, mobile methyl group and immobile alkyl chains:

5 $${\rm{S2}}={\rm{0.60}}\times {\rm{MM}}+{\rm{2.02}}\times {\rm{MA}}+{\rm{0.12}}\times {\rm{IA}}-{\rm{1.85}}$$

where MM is mobile methyl chains (-CH_3_ groups), MA is mobile alkyl chains (CnH_2_n groups, n >1) and IA is immobile alkyl chains (CnH_2_n groups, n >1).

The coefficient of determination (R^2^) between the true S2 and modeled S2 was 0.98, and the RMS (root mean square) error of prediction was ± 0.70 mg hydrocarbon per gram of rock. It was also, observed that the by adding additional structural parameters in the multiple regression model, the coefficient of determination (R^2^) between true vs. modeled S2 did not improve. This observation further suggests that the structural parameters used in the model are the primary contributors of hydrocarbon potential.

### Model validation

**Figure 4:**
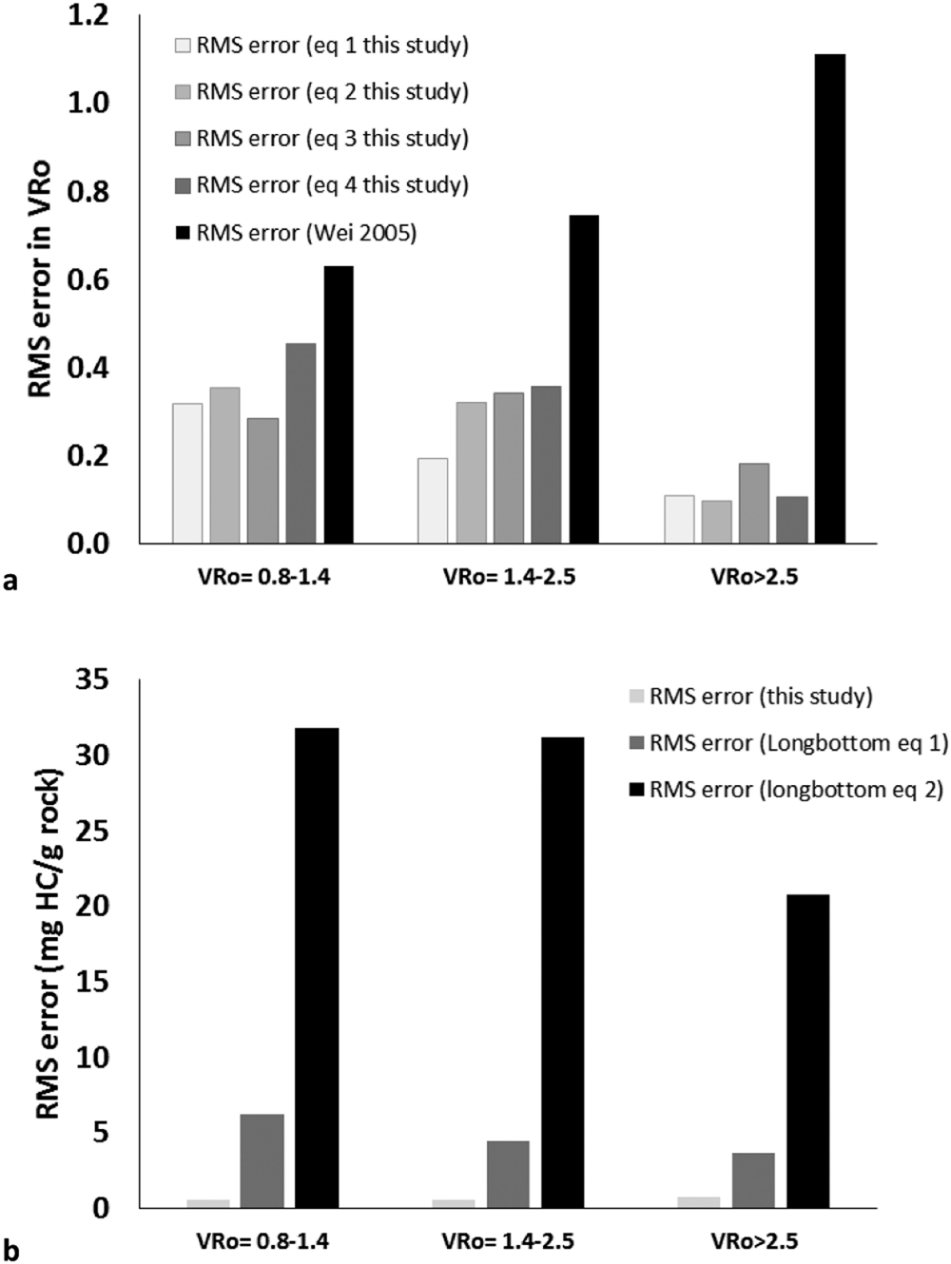
Comparison of RMS error of (**a**) thermal maturity and (**b**) HC generation, determined using models proposed in this study with previous kerogen models.

To validate the model proposed for determining thermal maturity, we determined the VRo values from the four equations proposed in this study and compared it with the true VRo values and values determined using previous regression model proposed by Wei *et al*.^26^ (shown in Table 2 of the Supplementary Information). The VRo values predicted using the newly build regression model were comparable to the true values with RMS error in VRo for all the samples were 0.21, 0.26, 0.26, 0.31 using equations 1–4 respectively. However, the VRo value predicted using equations proposed by Wei *et al*.^26^ underestimated the true values with a total RMS error 0.91 in VRo values (Fig. 4a, Table 2 of the Supplementary Information). The RMS error for predicting VRo in different maturity ranges using the proposed equations were also significantly less than the previously proposed model (Fig. 4a, Table 2 of the Supplementary Information).

To validate the model proposed for determination of hydrocarbon potential, we determined the total amount of HC generated using the modeled values of S2 and compared it with the true amount of HC generated. The true amount of total HC generated (in mg) per gram of rock is determined by equation S2_net_ = S2_org_ − S2_pd_ (org stands for original and pd for the present day). S2_org_ is calculated by the dividing the TOC_org_ by HI_org_. TOC_org_ is calculated using equation given by Peters *et al*.^42^:

$${{\rm{TOC}}}_{{\rm{org}}}=83.33\,({{\rm{HI}}}_{{\rm{pd}}})({{\rm{TOC}}}_{{\rm{pd}}})/{{\rm{HI}}}_{{\rm{org}}}\,(1-f)(83.33-{{\rm{TOC}}}_{{\rm{pd}}})+({{\rm{HI}}}_{{\rm{pd}}})({{\rm{TOC}}}_{{\rm{pd}}})$$

where *f* = 1 − [HI_pd_ (1200 − (HI_org_/1 − PI_org_)/HI_org_ (1200 − (HI_pd_/I − PI_pd_] HI_org_ obtained for immature Marcellus shale is approximately 250 mg HC/g rock^46^. Similar HI_org_ were obtained from HI vs. VRo plot (Y-intercept of the curve Fig. 5 in Supplementary Information). PI_org_ is assumed to be 0.02 for the most immature source rocks^42^.The S2 values modeled in this study are the S2_present day_. The total amount of HC generated calculated using the true S2 values and modeled values are shown in Table 3 in the Supplementary Information. We compared these values with true amount of HC generated and HC generated using previous regression models (Table 3 in Supplementary Information). The amount of HC generated predicted using the newly build regression model were comparable to the true values with RMS error for all the samples 0.70 mg HC/g rock. However, the amount of HC generated predicted using the two equations proposed by Longbottom *et al*.^28^ underestimated the true values with RMS error 3.93 mg HC/g rock and 27.05 mg HC/g rock (Fig. 4b, Table 3 in Supplementary Information). The RMS error for predicting HC generated in different maturity ranges using the proposed model was also significantly less than the previously proposed model (Fig. 4b, Table 3 in Supplementary Information). The higher RMS error of older models could be attributed to the fact that they (1) utilized kerogen derived from shales that were all below VRo <1 and, (2) used shale samples acquired from different basins where the structure of kerogen might vary significantly due to variations in sources of OM and depositional conditions.

The major strength of new models proposed in this study is that they were generated using kerogen extracted from samples across the entire maturity range of hydrocarbon generation, and therefore more accurately represent the source rock in mature shale plays like Marcellus as compared to older models. Future work will focus on acquiring samples from other shales to test the efficacy of our models. It is also plausible that for more accurate estimation of HC generative potential and maturity, similar kind of models need to be developed for individual plays instead of utilizing generalized models.

## Methods

### 13C solid-state NMR analysis

Solid-state NMR experiments were performed on a Bruker Advance III 400 spectrometer operating at 400-MHz 1H and 100-MHz ^13^C frequencies at Environmental NMR Service at Old Dominion University in Norfolk, Virginia, USA. The ^13^C chemical shifts were referenced to tetramethyl silane, using the COO resonance of glycine in the α-modification at 176.46 ppm as a secondary reference. Quantitative ^13^C NMR spectra for all the kerogen samples were acquired using the high-spinning speed multi-ramped amplitude cross polarization/magic angle spinning technique developed by Johnson and Schmidt-Rohr^47^. This multiple-cross polarization (multiCP) technique is a simple, robust way to obtain quantitative solid-state ^13^C NMR spectra of kerogen, with good signal-to-noise ratio. The spectra were measured at a spinning speed of 14 kHz, where spinning sidebands are fairly small (<3%) and have little overlap with center bands. ^13^C multiCP/MAS with dipolar dephasing was performed under the same conditions as for ^13^C multiCP/MAS but combined with a dipolar dephasing time of 68 μs^40^ to differentiate nonprotonated C from total C and to determine fractions of mobile groups (with no restricted rotation). The relative proportion of different carbon chains as shown in Supplementary Table 1 were obtained from ^13^C NMR spectrum using an NMR peak fitting program *TopSpin* (area of spectra from 0–240 ppm was considered to be 100% as detailed in Agrawal and Sharma^19^).

### SRA analysis

Approximately 80 mg of powdered (200 mesh) shale sample was weighed into a SRA crucible and placed in the autosampler and held isothermally at 300 °C for 3 minutes. The free hydrocarbons are volatilized during this isothermal heating which is quantitatively detected by the FID detector and reported as milligrams (mg) of S1 per gram of rock. The free CO_2_ is simultaneously liberated which is detected by the IR cell and reported as milligrams (mg) of S3 per gram of rock. The temperature is increased after the isothermal period at the rate of 25 °C/minute until 600 °C. Pyrolytic degradation of the kerogen takes place between 300 °C and 600 °C generating HCs. These hydrocarbons are also detected by the FID are labeled as S2, reported as mg of S2 per gram of rock. The temperature at the peak of S2 generation is known as Tmax. It is used to estimate the thermal maturity of shale samples. Vitrinite reflectance is calculated using equation (VRo) = 0.018 × Tmax −7.16^48^. Residual carbon is also measured by SRA and is reported as S4. TOC of the sample is calculated using the equation 0.1 × [0.082 × (S1 + S2) + S4], in wt %. After every five sample WFT Source Rock Standard 533 (P/N 810-141) was run. SRA analysis was performed at the National Energy Technology Laboratory and at IsoBioGEM lab in Morgantown.

## Electronic supplementary material

Supplementary Information
