# Supplementary material for: Improved Kerogen Models for Determining Thermal Maturity and Hydrocarbon Potential of Shale
Source: Sci Rep. 2018 Nov 30;8:17465. doi: 10.1038/s41598-018-35560-8 (PMC6269451; doi:10.1038/s41598-018-35560-8)
Supplement: Supplementary file 1 — Supplementary Information [file 41598_2018_35560_MOESM1_ESM.pdf]

IMPROVED KEROGEN MODELS FOR DETERMINING THERMAL MATURITY AND HYDROCARBON POTENTIAL OF SHALES

Vikas Agrawal[1], Shikha Sharma[1]*

[1]Department of Geology and Geography, West Virginia University, Morgantown, WV, United States.

*shikha.sharma@mail.wvu.edu[1]

Supplementary information

Table 1 Structural parameters obtained for kerogen samples using 13C solid state NMR (^{13}C multiCP/MAS with and without dipolar dephasing)

Structural parameters	Chemical Shift	BG-1 UM**	BG-1 LM**	WV-7 UM**	WV-7 LM**	MW-1 UM	MW-1 LM	WV-6 UM**	WV-6 LM**	BL-3H UM	BL-3H LM	MIP-3H UM	MIP-3H LM	MIP 3H MT	MIP 3H MM	MIP 3H MO
Total aliphatic	0-90	0.30	0.25	0.26	0.27	0.17	0.15	0.15	0.14	0.09	0.13	0.06	0.07	0.10	0.07	0.09
Alkyl	0-45	0.26	0.20	0.21	0.23	0.14	0.13	0.08	0.10	0.07	0.08	0.05	0.06	0.07	0.06	0.06
alkyl (0-20)	0-20	0.07	0.05	0.06	0.07	0.04	0.03	0.03	0.04	0.03	0.03	0.02	0.02	0.04	0.03	0.02
Alkyl (20-45)	20-45	0.18	0.15	0.15	0.16	0.10	0.10	0.05	0.06	0.03	0.04	0.04	0.04	0.04	0.03	0.04
Mobile methyl*	(0-20)	0.03	0.03	0.03	0.03	0.01	0.02	0.00	0.01	0.02	0.00	0.00	0.01	0.02	0.01	0.02
Mobile and quaternary alkyl*	(20-45)	0.05	0.04	0.04	0.03	0.01	0.02	0.01	0.01	0.00	0.01	0.01	0.02	0.02	0.01	0.02
Immobile methyl*		0.04	0.02	0.03	0.04	0.04	0.01	0.03	0.03	0.02	0.03	0.02	0.02	0.02	0.01	0.00
Immobile alkyl (20-45)*		0.13	0.11	0.11	0.12	0.08	0.08	0.04	0.05	0.03	0.03	0.02	0.02	0.02	0.02	0.03
Methoxyl and amine*	(45-60)	0.01	0.02	0.02	0.02	0.01	0.01	0.02	0.01	0.01	0.02	0.00	0.00	0.01	0.00	0.01
Methoxyl*	(45-60)	0.00	0.00	0.00	0.00	0.00	0.00	0.00	0.00	0.01	0.01	0.00	0.00	0.01	0.00	0.01
O and O2 substituted alkyl	(60-90)	0.04	0.03	0.03	0.02	0.01	0.01	0.05	0.03	0.02	0.03	0.00	0.00	0.02	0.01	0.02
Total aromatic	(90-165)	0.67	0.70	0.76	0.75	0.79	0.82	0.85	0.89	0.90	0.86	0.89	0.92	0.89	0.90	0.90
Aromatic bridgehead*	(90-135)	0.15	0.25	0.30	0.28	0.19	0.22	0.33	0.37	0.23	0.34	0.32	0.49	0.33	0.37	0.38
Protonated aromatic	(90-135)	0.35	0.28	0.33	0.35	0.41	0.43	0.41	0.44	0.53	0.34	0.39	0.23	0.43	0.36	0.37
Alkyl substituted aromatic	135-150	0.15	0.16	0.13	0.12	0.16	0.15	0.10	0.09	0.13	0.15	0.16	0.17	0.12	0.15	0.14
Oxygen substituted aromatic	150-165	0.02	0.02	0.00	0.00	0.02	0.03	0.01	0.00	0.02	0.03	0.03	0.02	0.01	0.02	0.02
Carboxyl and amide*	(165-190)	0.05	0.03	0.00	0.00	0.03	0.00	0.01	0.00	0.01	0.01	0.02	0.02	0.01	0.01	0.01
Aldehyde and ketone	190-240	0.00	0.01	0.00	0.00	0.02	0.02	0.00	0.00	0.01	0.00	0.03	0.00	0.00	0.01	0.00
Cn'		2.05	1.59	2.00	2.27	1.03	1.00	1.47	1.50	0.74	0.87	0.36	0.40	0.85	0.47	0.62
SP2 to SP3 carbon		2.34	2.92	2.91	2.77	5.03	5.76	5.78	6.54	9.87	6.94	16.48	13.90	8.62	13.18	10.76

*calculated using dipolar dephasing method

**values from *Agrawal and Sharma, 2018*[19]

Table 2 True vs Modeled VRo

Sample	True VRo	Modeled VRo using eq 1	Modeled VRo using eq 2	Modeled VRo using eq 3	Modeled VRo using eq 4	Modeled VRo using Wei et al., 2005
**VRo= 0.8-1.4**						
BG-1 UM	1	0.74	0.56	0.44	0.51	1.06
BG-1 LM	0.81	1.16	1.09	0.85	1.22	0.71
WV-7 UM	1.4	1.27	1.13	1.39	1.07	0.56
WV-7 LM	1.4	0.95	1	1.3	0.84	0.47
**RMS error**		**0.32**	**0.35**	**0.29**	**0.46**	**0.63**
**VRo= 1.4-2.5**						
MW-1 UM	1.49	1.76	1.98	1.74	1.98	0.88
MW-1 LM	1.61	1.76	1.93	2.14	2.09	1.16
WV-6 UM	2.5	2.71	2.76	2.4	2.71	1.42
WV-6 LM	2.5	2.4	2.54	2.84	2.51	1.8
**RMS error**		**0.19**	**0.32**	**0.34**	**0.36**	**0.75**
**VRo>2.5**						
BL-3H UM	2.93	2.86	3	2.99	2.92	1.94
BL-3H LM	2.96	2.76	2.81	2.52	2.75	1.53
MIP-3H UM	2.94	2.96	2.92	2.88	3.06	1.84
MIP-3H LM	2.98	3	2.88	3.11	2.95	2.04
MIP 3H MT	2.92	3.07	2.96	2.8	2.8	1.76
MIP 3H MM	2.96	3.07	2.99	3	3.01	1.92
MIP 3H MO	2.97	2.9	2.82	2.94	2.95	1.94
**RMS error**		**0.11**	**0.1**	**0.18**	**0.11**	**1.11**
**Net RMS error**		0.21	0.26	0.26	0.31	0.91

Table 3 True vs Modeled amount of HC generated

Sample	True HC	Modeled HC generated<br>This study	Modeled HC generated<br>Longbottom eq 1	Modeled HC generated<br>Longbottom eq 2
**VRo= 0.8-1.4**				
BG-1 UM	6.86	7.37	-0.54	-5.09
BG-1 LM	25.1	25.91	28.62	-4.85
WV-7 UM	7.85	7.53	-1.51	-22.51
WV-7 LM	25.27	24.69	23.82	-20.51
**RMS error**		**0.58**	**6.26**	**31.85**
**VRo= 1.4-2.5**				
MW-1 UM	24.2	24.47	20.67	4.11
MW-1 LM	21.18	20.2	18.1	4.86
WV-6 UM	11.01	11.96	3.43	-9.7
WV-6 LM	27.45	27.04	26.07	-25.37
**RMS error**		**0.73**	**4.51**	**31.18**
**VRo>2.5**				
BL-3H UM	16.49	17.4	12.78	-0.6
BL-3H LM	28.27	27.95	26.75	4.05
MIP-3H UM	12.78	13.53	8.7	-2.65
MIP-3H LM	26.8	25.47	29.72	0.3
MIP 3H MT	9.71	10.18	3.06	-4.38
MIP 3H MM	20.21	19.9	19.3	-3.78
MIP 3H MO	16.28	15.82	13.2	-4.51
**RMS error**		**0.74**	**3.7**	**20.79**
**Net RMS error**		**0.7**	**3.93**	**27.05**

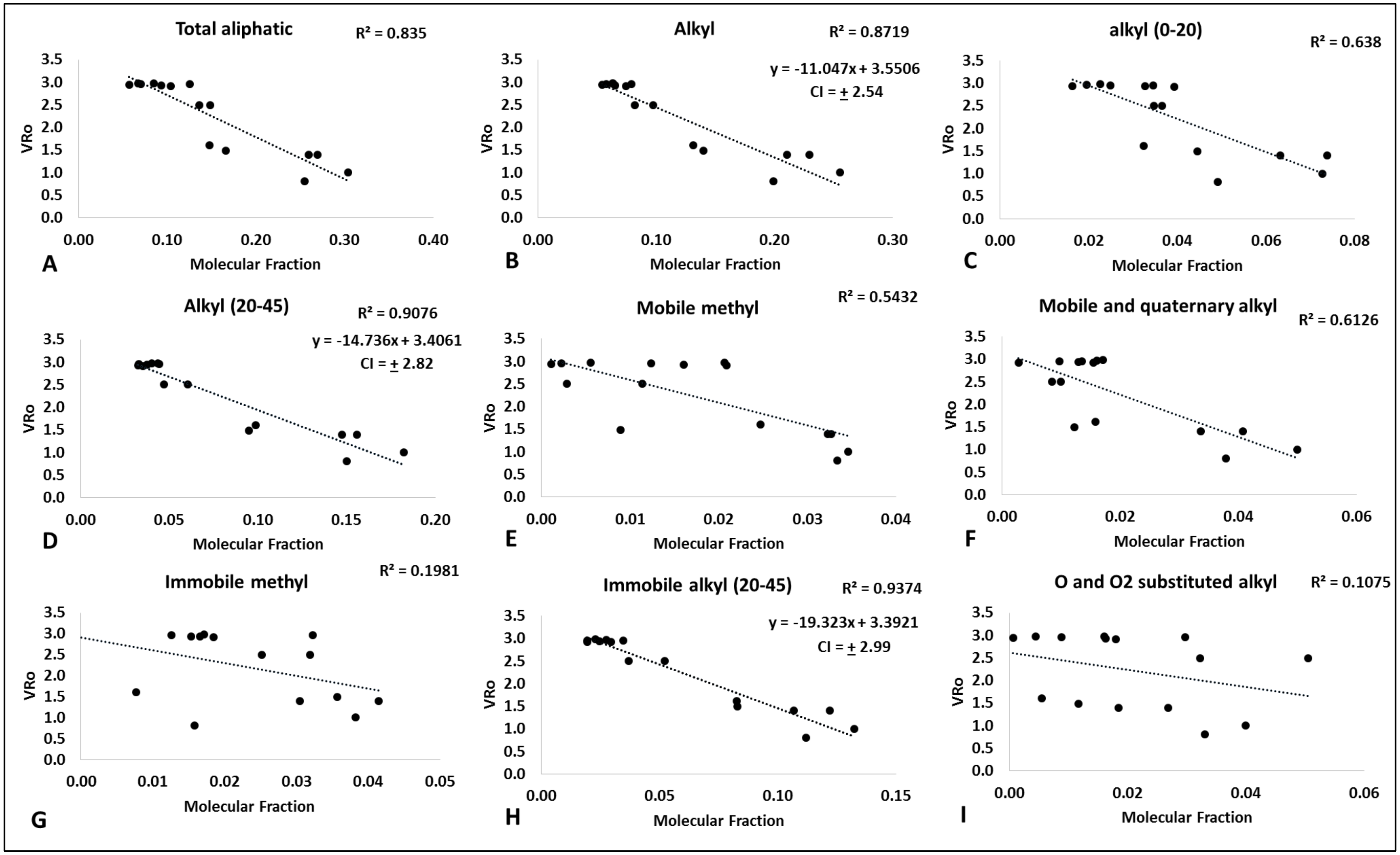

Figure.1 Correlation of fraction of different aliphatic structural parameters of kerogen (X axis) vs Vtrinite reflectance, VRo (Y axis). Correlation coefficient (R^2) is indicated for each cross plot. CI indicated 95% confidence interval of slope.

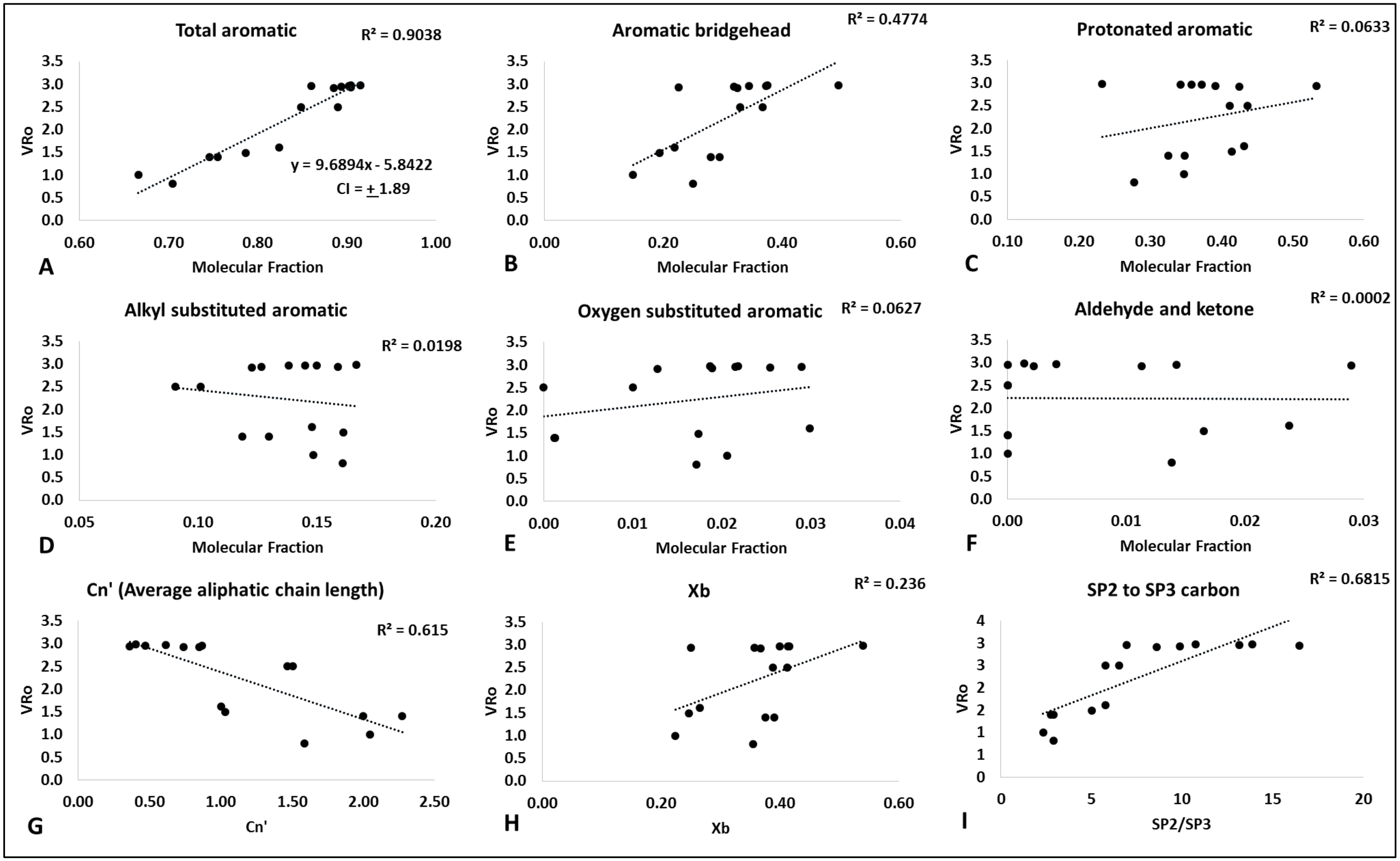

Figure.2 Correlation of fraction of different aromatic and lattice structural parameters of kerogen (X axis) vs Vtrinite reflectance, VRo (Y axis). Correlation coefficient (R^2) is indicated for each cross plot.

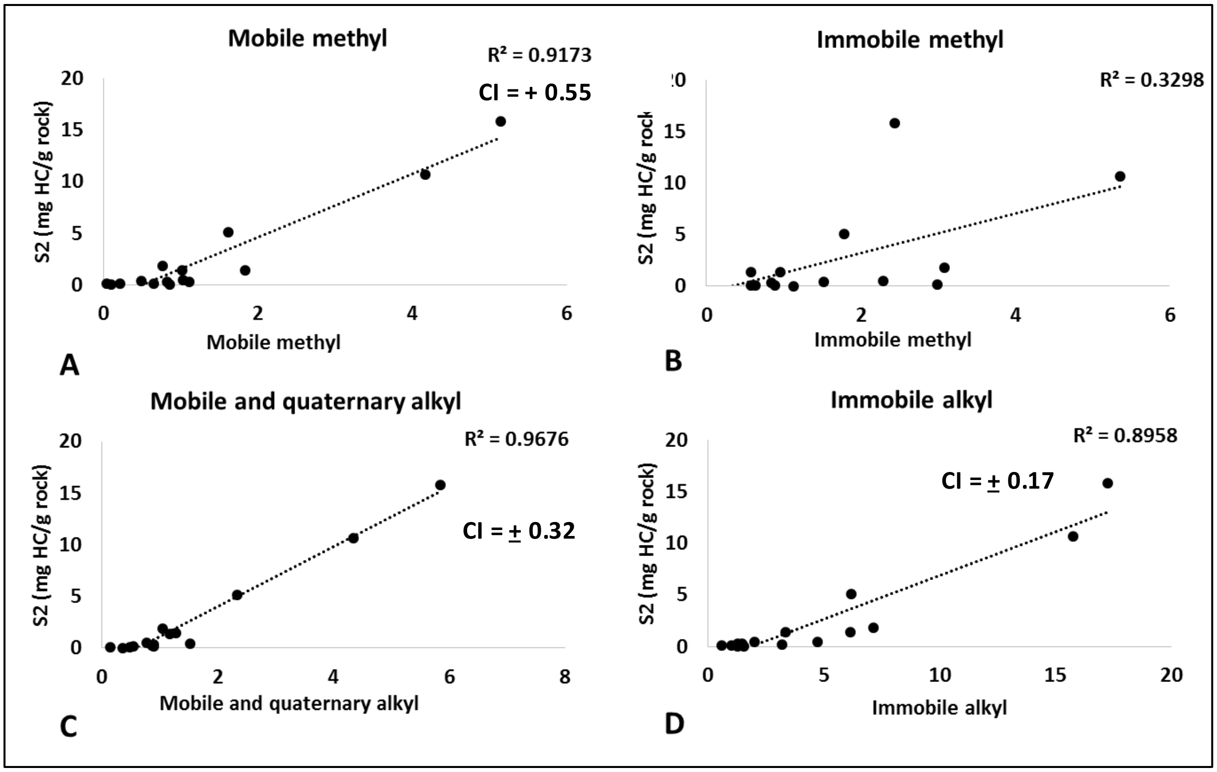

Figure.3 Correlation of fraction of different aliphatic structural parameters of kerogen, in mg/g rock (X axis) vs HC generative potential, S2, in mg HC/g rock (Y axis). Correlation coefficient (R^2) is indicated for each cross plot. CI indicated 95% confidence interval of slope.

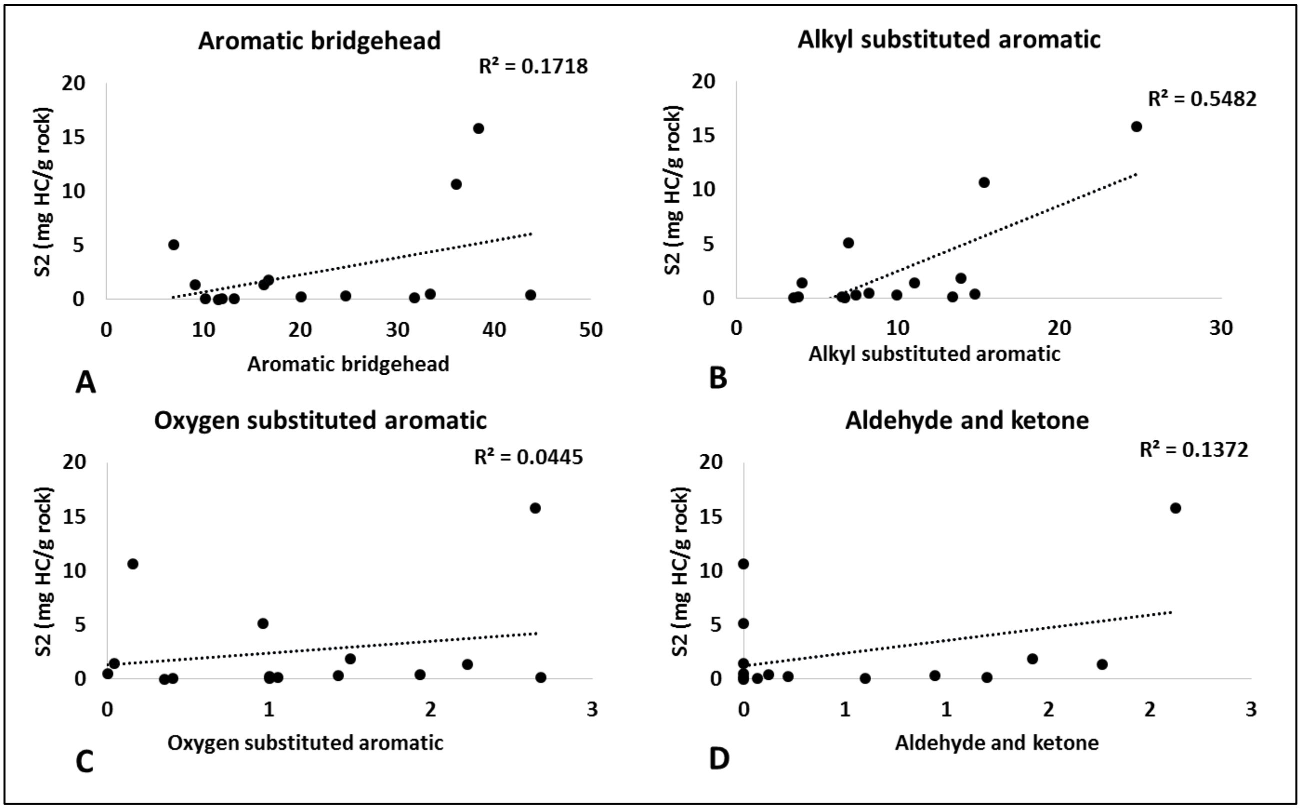

Figure.4 Correlation of fraction of different aromatic parameters of kerogen, in mg/g rock (X axis) vs HC generative potential, S2, in mg HC/g rock (Y axis). Correlation coefficient (R^2) is indicated for each cross plot.

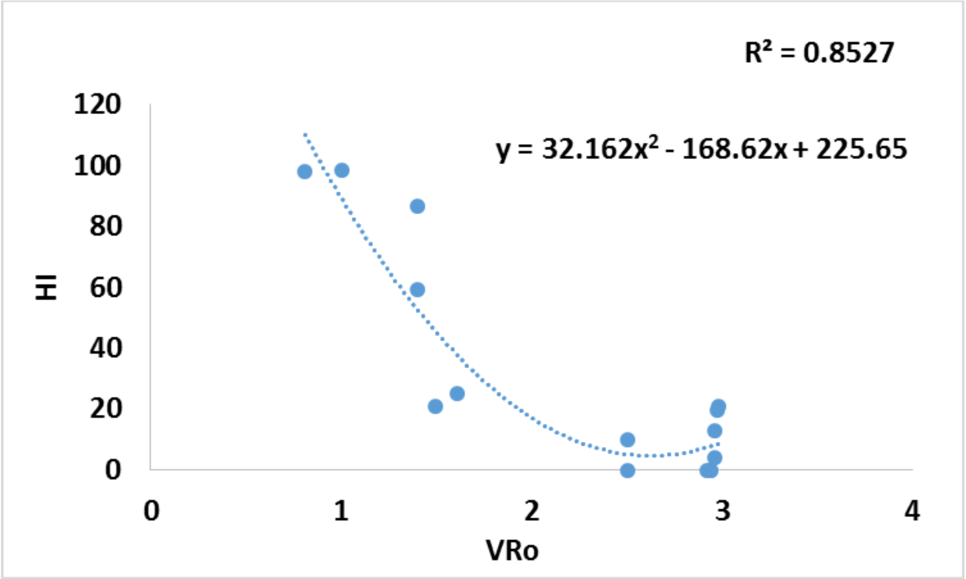

Figure.5 HI vs VRo cross plot. The Y intercept of the curve represents HI_{org}
